# Feature selection with the Fisher score followed by the Maximal Clique Centrality algorithm can accurately identify the hub genes of hepatocellular carcinoma

**DOI:** 10.1038/s41598-019-53471-0

**Published:** 2019-11-21

**Authors:** Chengzhang Li, Jiucheng Xu

**Affiliations:** 1 College of Life Science, Henan Normal University, Xinxiang, 453007 Henan Province China; 2 Engineering Lab of Intelligence Business & Internet of Things, College of Computer and Information Engineering, Henan Normal University, Xinxiang, 453007 Henan Province China; 3 State Key Laboratory Cultivation Base for Cell Differentiation Regulation, Henan Normal University, Xinxiang, 453007 Henan Province China; 4 Department of Physiology and Neurobiology, School of Basic Medical Sciences, Xinxiang Medical University, Xinxiang, 453003 Henan Province China

**Keywords:** Microarray analysis, Cancer, Targeted therapies, Predictive markers, Applied mathematics

## Abstract

This study aimed to select the feature genes of hepatocellular carcinoma (HCC) with the Fisher score algorithm and to identify hub genes with the Maximal Clique Centrality (MCC) algorithm. Gene Ontology (GO) and Kyoto Encyclopedia of Genes and Genomes (KEGG) enrichment analysis was performed to examine the enrichment of terms. Gene set enrichment analysis (GSEA) was used to identify the classes of genes that are overrepresented. Following the construction of a protein-protein interaction network with the feature genes, hub genes were identified with the MCC algorithm. The Kaplan–Meier plotter was utilized to assess the prognosis of patients based on expression of the hub genes. The feature genes were closely associated with cancer and the cell cycle, as revealed by GO, KEGG and GSEA enrichment analyses. Survival analysis showed that the overexpression of the Fisher score–selected hub genes was associated with decreased survival time (P < 0.05). Weighted gene co-expression network analysis (WGCNA), Lasso, ReliefF and random forest were used for comparison with the Fisher score algorithm. The comparison among these approaches showed that the Fisher score algorithm is superior to the Lasso and ReliefF algorithms in terms of hub gene identification and has similar performance to the WGCNA and random forest algorithms. Our results demonstrated that the Fisher score followed by the application of the MCC algorithm can accurately identify hub genes in HCC.

## Introduction

Gene microarray technology, a prospective tool for the classification, diagnosis and aggressiveness prediction of cancer, provides valuable information in understanding the underlying mechanism of multiple cancers^1–4^. The data obtained from microarray experiments, such as leukaemia datasets and breast cancer datasets^5,6^, are often used for feature selection in machine learning. In comparison with a large number of genes, the training datasets usually have a very small sample size for classification. The limitations of training data constitute a great challenge to certain classification methodologies^7^. Gene expression datasets with a large number of variables and only a small number of samples are normally referred to as having the curse of dimensionality in feature selection^8^. The prediction performance of feature selection highly depends on the quality and the size of the gene dataset^9^. However, some of the gene expression datasets, such as the Wisconsin breast cancer database^10^, were constructed approximately thirty years ago and may have defects due to the limitation of instrument performance at that time. In addition, less information in these older datasets may lead to poor feature selection performance. Therefore, the establishment of updated datasets is necessary for the development of feature selection. A large number of microarray gene expression datasets are available in the Gene Expression Omnibus (GEO) database and are updated regularly. GEO microarray datasets are also normally characterized by a small number of samples with high dimensionality. The integration of independent GEO datasets published in recent years can significantly enlarge the sample size, which may be helpful to combat the curse of dimensionality.

Feature selection, one of the vital and repeatedly used machine learning techniques in data mining, is the selection of a subset of the most pertinent features for use in the process of model construction^11^. In other words, feature selection is generally regarded as an optimization problem with the purpose of maximizing the classification accuracy with relatively fewer features^12,13^. To achieve this goal, irrelevant and redundant features of raw datasets are usually eliminated with the application of feature selection^14^. Since the 1970s, feature selection techniques have been widely employed in a variety of fields, such as protein structural class prediction^15^, classification of traced neurons^16^, text classification^17^, acoustic event recognition^18^ and gene expression data classification^19^. Feature selection techniques are also used to select marker genes of cancer that affect the classification accuracy^7^. Despite important advances that have been achieved in the microarray-based molecular classification of tumours, it is far from application in clinical practice^20,21^. To date, several feature selection algorithms, such as the Fisher score, Lasso, ReliefF and random forest algorithms, have been employed in the selection of feature genes^22–24^. Previous studies have demonstrated that the Fisher score has good performance in feature gene selection^21^.

In this study, we aimed to develop a hepatocellular carcinoma (HCC) hub gene identification method via the analysis of protein-protein interaction (PPI) networks. To build the PPI network, several individual genes that contribute to the classification of HCC are needed. Unlike some other feature selection algorithms, such as principal component analysis (PCA), in which the selected features are a combination of some raw features, the Fisher score algorithm selects each gene independently based on their scores under the Fisher criterion, which eventually leads to a subset of the most representative individual genes^25,26^. Therefore, this algorithm may be an appropriate method for the feature selection of high dimensional gene expression profile data. To date, this algorithm has received less attention in the field of HCC feature gene selection.

We constructed and integrated an HCC gene expression dataset from five independent HCC gene expression datasets and utilized the Fisher score algorithm to select feature genes for HCC. Gene Ontology (GO) and Kyoto Encyclopedia of Genes and Genomes (KEGG) enrichment analysis^27^ were performed to examine the molecular functions (MFs), cellular components (CCs), biological processes (BPs) and pathways of the selected feature genes. Gene set enrichment analysis (GSEA)^28^ was carried out to evaluate the feature selection performance of the Fisher score algorithm at the gene set level. To explore the association between the feature genes, the Search Tool for the Retrieval of Interacting Genes/Proteins (STRING) database was applied to establish the PPI^27^ network, which was then analysed with the Maximal Clique Centrality (MCC)^29^ method to select the top ten hub genes of HCC. The Kaplan–Meier plotter was utilized to assess the role of the selected hub genes in liver cancer prognosis. To further evaluate the performance of the Fisher approach, weighted gene co-expression network analysis (WGCNA), one of the most widely used hub gene identification approaches, along with the Lasso, ReliefF and random forest algorithms, were used as comparison algorithms.

## Results

### Integrated dataset of liver microarray gene expression profiles

A significant batch effect was identified in the raw data of 5 independent gene expression datasets (Supplementary Fig. 1(a)). After correcting the batch effect with the removeBatchEffect function of the limma package, no batch effect was observed based on the observation of PCA components (Supplementary Fig. 1(b)). The ultimate integrated dataset after the correction of the batch effect has 396 samples and 54613 variables (probe IDs) and takes up 214 MB of computer memory (Supplementary Dataset 1). Each sample is labelled with both its GSE ID and GSM ID.

### Feature gene selection with the fisher score algorithm

Feature selection techniques can avoid the curse of dimensionality and thus enable the simplification of models, making interpreting experimental results easier for researchers. As one of the supervised feature selection methods, the Fisher score algorithm selects each feature independently in accordance with their scores. Here, Feature selection using the Fisher score algorithm results in a list of genes that are ranked by their importance. A previous study showed that approximately 1000 genes were biologically relevant to HCC^30^. Therefore, we also selected the top 1000 (Supplementary Dataset 2) feature genes with higher Fisher scores as the optimal feature subset for further analysis.

### GO and KEGG enrichment analysis

**Figure 1:**
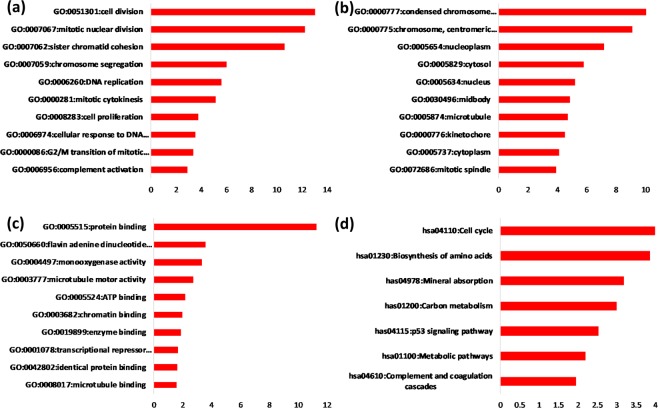
GO and KEGG enrichment analysis of feature genes in HCC. The numbers below each panel are reference P values (−log10). (**a**) GO biological process enrichment analysis of the feature genes in HCC. (**b**) GO cellular component enrichment analysis of the feature genes in HCC. (**c**) GO molecular function enrichment analysis of the feature genes in HCC. (**d**) KEGG pathway enrichment analysis of the feature genes in HCC.

A total of 37 significantly enriched BPs were observed in the current study, which involved many cancer-associated BPs, such as cell division, mitotic nuclear division, positive regulation of cell proliferation, cell proliferation, negative regulation of the apoptotic process, sister chromatid cohesion, DNA replication, regulation of the apoptotic process, the cell cycle, and the G2/M transition of the mitotic cell cycle (GO IDs: 0051301, 0007067, 0007062, 0008284, 0008283, 0043066, 0007062, 0006260, 0042981, and 0000086, respectively) (Fig. 1(a)). GO CCs were significantly enriched in the condensed chromosome kinetochore, chromosome, nucleoplasm, cytosol, nucleus, microtubule, kinetochore and so on (GO IDs: 0000777, 0000775, 0005654, 0005829, 0005634, 0005874 and 0000776, respectively) (Fig. 1(b)). The GO MFs were mainly enriched in protein binding, flavin adenine dinucleotide binding, microtubule motor activity, ATP binding, chromatin binding, enzyme binding, RNA polymerase II core promoter proximal region sequence-specific binding, and microtubule binding (GO IDs: 0008017, 0042802, 0003777, 0005524, 0003682, 0019899, 0001078, and 0008017, respectively) (Fig. 1(c)).

Figure 1(d) shows that the most significantly enriched KEGG pathway was the cell cycle (pathway ID: 04110), which is directly associated with cancer. In addition, the biosynthesis of amino acids, carbon metabolism, the p53 signalling pathway and metabolic pathways (pathway IDs: 01230, 01200, 04115, and 01100, respectively) were also closely linked to the progression of liver cancer.

### GSEA

**Figure 2:**
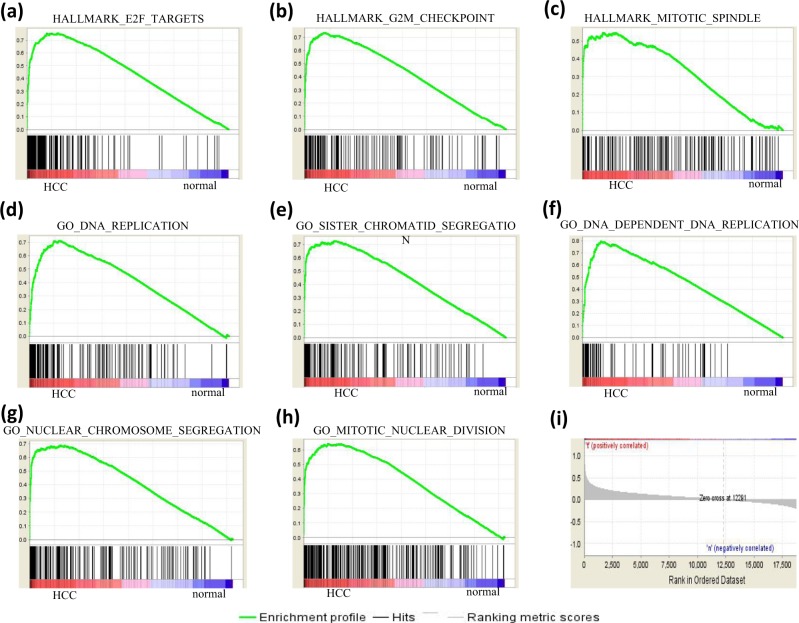
GSEA. (**a–c**) Displays the top 3 most upregulated gene sets of GSEA based on the hallmark gene sets. (**d–h**) shows the top 5 most upregulated gene sets of GSEA based on the GO gene sets. The enrichment score (NES), false discovery rate (FDR) and the normalized enrichment score are shown for each gene set. The bars at the bottom of the panels are the corresponding genes of certain gene sets. (**i**) Displays the relative location of genes in the ranked list.

To evaluate the feature selection performance of the Fisher score algorithm at the gene set level, GSEA was carried out based on hallmark gene sets and GO gene sets. Most of the parameters used for GSEA were set as default. The number of permutations was 1000, and the permutation type was gene set. The min and max size of the selected gene sets were 10 and 500, respectively. When performing GSEA based on hallmark gene sets, 18/50 gene sets were upregulated in the cancer phenotype, 11 gene sets were significant at false discovery rate (FDR) < 25%, and 9 gene sets were significantly enriched at nominal p value < 5%. The top 3 upregulated gene sets were E2F targets, G2M checkpoint and mitotic spindle (Fig. 2(a–c), (i)). For GSEA based on GO gene sets, 1105/3322 gene sets were upregulated in the cancer phenotype, 471 gene sets were significant at FDR < 25%, and 400 gene sets were significantly enriched at nominal p value < 5%. The most significantly enriched genes included DNA replication, sister chromatid segregation, DNA-dependent DNA replication, nuclear chromosome segregation, and mitotic nuclear segregation (Fig. 2(d–h,i)).

### PPI network establishment and hub gene identification

**Figure 3:**
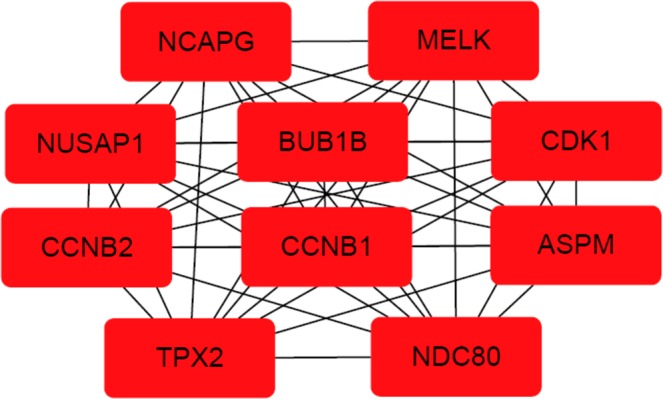
The PPI network of the top ten hub genes in HCC. The nodes represent the selected feature genes, and the edges represent the interactions between two genes.

A PPI network was constructed with all genes significantly enriched in BPs with the STRING database. A total of 365 nodes and 4326 edges were involved in the PPI network (Supplementary Fig. 2). After PPI network establishment, the PPI data were then imported into Cytoscape software. CytoHubba, an app of Cytoscape, is usually employed to predict important nodes or subnetworks in a given network based on several topological algorithms. Here, the top ten hub genes were selected based on the MCC algorithm in cytoHubba (Fig. 3). The results showed that the top ten genes contributing to HCC were ASPM, MELK, CCNB1, NDC80, BUB1B, NCAPG, CDK1, NUSAP1, CCNB2 and TPX2.

### Survival analysis

**Figure 4:**
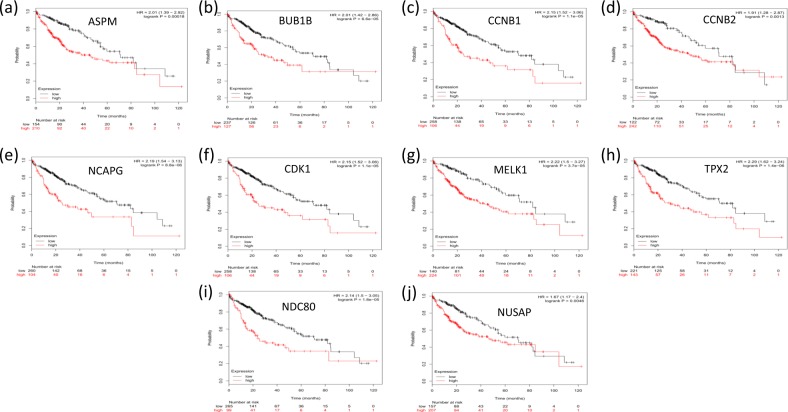
Kaplan–Meier survival analysis of the hub genes selected with the Fisher score. (**a–j**) shows a Kaplan–Meier plot of the top ten HCC hub genes. In comparison with that in normal subjects, the overexpression of these hub genes in HCC patients was associated with a significant reduction in overall survival time (P < 0.05).

The Kaplan–Meier plotter was utilized to assess the effect of the top ten hub genes on liver cancer prognosis. A total of 364 liver cancer cases were available for overall survival analysis. Our study showed that the overexpression of the hub genes selected with the Fisher score was correlated with a significant reduction in the overall survival time of liver cancer patients (P < 0.01, Fig. 4).

### Comparison of the fisher score with other algorithms

**Figure 5:**
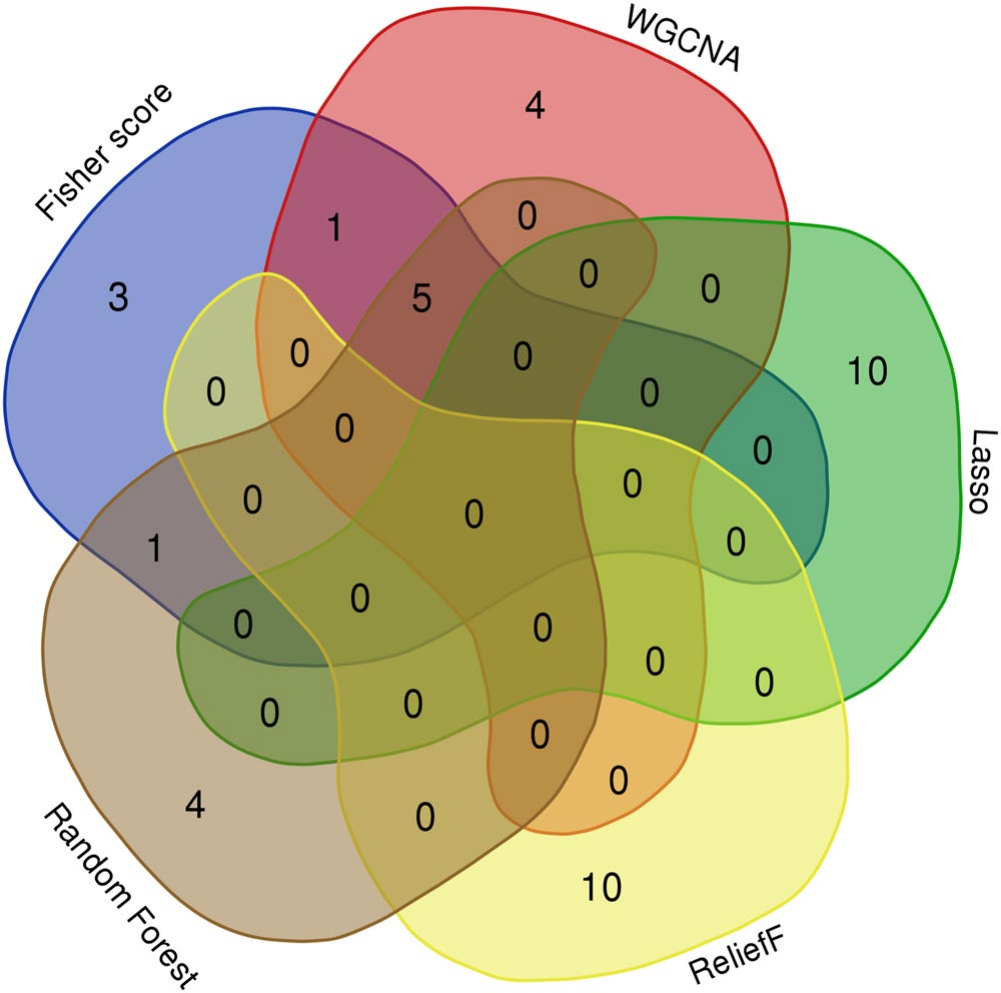
A Venn diagram showing the overlapping hub genes selected with the Fisher score, WGCNA, Lasso, ReliefF and random forest algorithms.

To assess the effect of the Fisher score algorithm, WGCNA, Lasso, ReliefF and random forest were also employed to select feature genes for HCC with the same integrated dataset followed by the identification of hub genes with the MCC algorithm. A Venn diagram (Fig. 5) showed that six hub genes selected with the Fisher algorithm were the same as those selected with the WGCNA or random forest algorithms and that no common genes were selected between the Fisher score algorithm and the Lasso or ReliefF algorithms.

The role of the selected hub genes with the abovementioned algorithms was also subject to survival analysis with the Kaplan–Meier plotter. Since 6 hub genes selected by WGCNA or random forest were the same as those selected by the Fisher algorithm (Fig. 4), we thus displayed only the unique genes selected by WGCNA or random forest.

**Figure 6:**
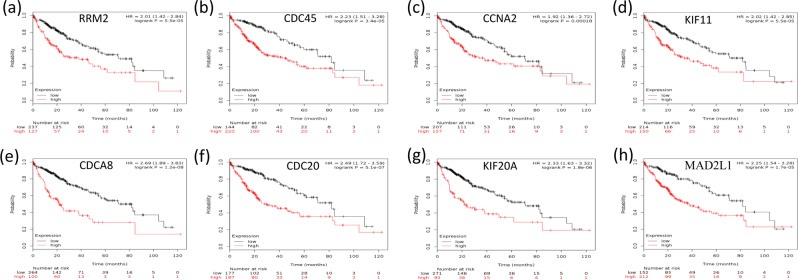
Kaplan–Meier survival analysis of hub genes selected with WGCNA and random forest. (**a–d**) shows that the unique hub genes selected with WGCNA, and (**e–h**) are the unique hub genes selected with random forest. The overexpression of all the unique hub genes was significantly correlated with a decrease in the survival time of HCC patients (P < 0.05).

**Figure 7:**
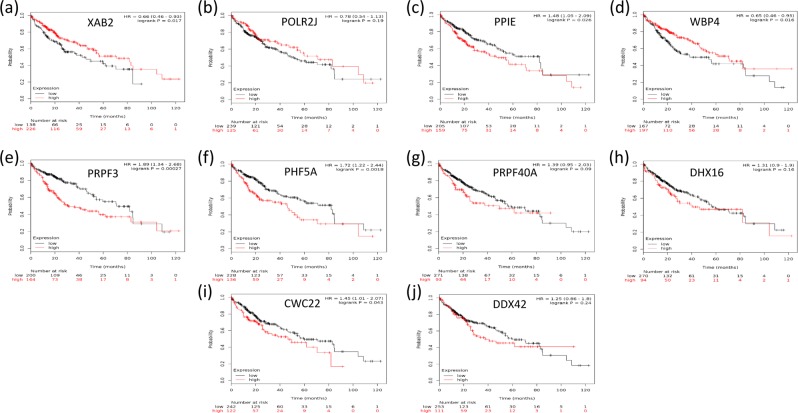
Kaplan–Meier survival analysis of hub genes selected with Lasso. Most of the hub genes selected with Lasso were either associated with increased survival time ((**a,d**), P < 0.05) or had no relationship with survival time (P > 0.05, (**b,g,h,j**)). Only four hub genes ((**c,e,f,i**), P < 0.05) were linked to the poor prognosis of HCC.

**Figure 8:**
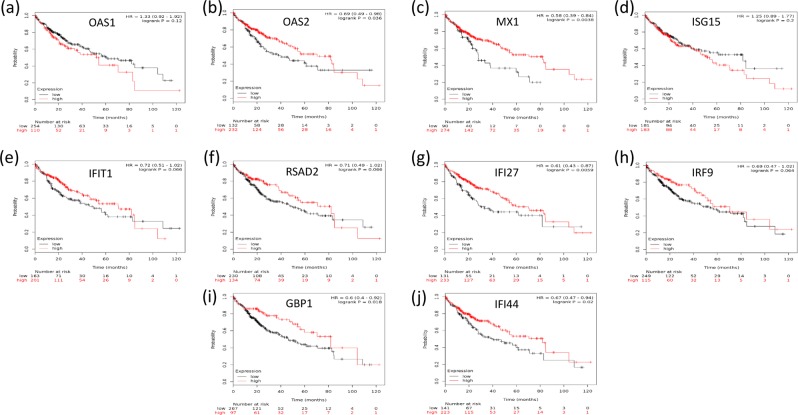
Kaplan–Meier survival analysis of hub genes selected with ReliefF. One-half of the hub genes were associated with increased survival time in HCC patients (P < 0.05, (**b,c,g,I,j**)), and the other half had no effect on survival time (P > 0.05, (**a,d,e,f,h**)).

Survival analysis showed that the overexpression of the unique hub genes selected with WGCNA (Fig. 6(a–d), P < 0.05) or with random forest (Fig. 6(e–h), P < 0.05) were significantly correlated with a decrease in the survival time of HCC patients. In contrast, most hub genes selected with Lasso were either associated with an increased survival time (Fig. 7(a,d), P < 0.05) or had no relationship with the survival time (P > 0.05, Fig. 7(b,g,h,j)). Only four hub genes (Fig. 7(c,e,f,i), P < 0.05) were linked to the poor prognosis of HCC. Regarding the hub genes selected with the ReliefF algorithm, half were involved in increased survival time (P < 0.05, Fig. 8(b,c,g,i,j)), and the other half had no effect on the survival time (P > 0.05, Fig. 8(a,d,e,f,h)).

## Discussion

Cancer encompasses many diseases that are characterized by the spread of abnormal cells and uncontrolled growth^31^. The overall occurrence of cancer is rapidly growing globally. An estimated 18.1 million new cancer cases and 9.6 million cancer deaths occurred in 2018^32^. Among all cancers, HCC is the fifth most frequently diagnosed cancer, ranking as the third leading cause of cancer-related death^33^. Currently, the main HCC treatment strategies include surgical resection, microwave ablation, radiofrequency ablation and transcatheter arterial chemoembolization (TACE)^34,35^. Regarding the prospects of a cure, surgical resection is believed to have a definitive curative effect^36^. However, most HCC cases are detected in advanced stages with the invasion of major blood vessels, obvious extrahepatic metastases or poor liver function, making them unfit for surgical resection. A prospective study conducted from December 2009 to December 2010 indicated that recurrent HCC patients are ineligible for percutaneous ablation^37^. Conventional TACE is fit for advanced HCC treatment and involves the delivery of chemotherapeutic agents that target cancer cells, which may cause the release of cytotoxic agents^38^ and acute pancreatitis^39^, as those drugs do not target the expression of the hub genes of cancer. For this reason, discovering the hub genes of advanced HCC is necessary for the purpose of treatment with specific drugs.

In this study, the feature genes that contribute to the occurrence of HCC were selected using the Fisher score algorithm. GO and KEGG enrichment analysis was performed to interpret the functions and pathways of the feature genes. The enriched BPs included cell division, mitotic nuclear division, positive regulation of cell proliferation, cell proliferation, negative regulation of the apoptotic process, sister chromatid cohesion, DNA replication, regulation of the apoptotic process, the cell cycle, and the G2/M transition of the mitotic cell cycle. These processes are typically representative features of HCC progression. The selected genes with the Fisher score algorithm were matched with the genes implicated in the abovementioned complex process of cancer development, indicating that the Fisher score algorithm is an effective method for selecting feature genes in HCC. The effectiveness of the Fisher score algorithm was further confirmed by GO CCs and GO MFs, which were related to cell proliferation and division. The top enriched KEGG pathway was the cell cycle-related signalling cascade, which contributes to the molecular mechanisms of hepatocarcinogenesis. Moreover, other enriched pathways, such as the biosynthesis of amino acids, carbon metabolism, p53 signalling and metabolism, are also associated with HCC proliferation and progression. Hence, the Fisher score algorithm is very efficient in feature gene selection. GSEA confirmed that the proliferation-related genes showed significant differences between the HCC and normal states.

A PPI network was established with the STRING database. With the application of the Cytoscape app cytoHubba, the top ten hub genes contributing to HCC were predicted and are as follows: ASPM, MELK, CCNB1, NDC80, BUB1B, NCAPG, CDK1, NUSAP1, CCNB2 and TPX2. Traditionally, the identification of biomarkers is mainly based on the metabolism of a pharmaceutical agent or the biology of the tumour and surrounding environment as performed in biological experiments^40^. Evidence from previous studies supports the effectiveness of the Fisher score algorithm in gene identification. Reverse transcription-PCR assays showed that ASPM is a marker for early recurrence and vascular invasion and that ASPM overexpression is correlated with poor prognosis in hepatocellular carcinoma^41^. The role of ASPM, MELK, NUSAP1, CCNB2 and NCAPG in HCC was validated by predictions performed with other bioinformatic tools as well as by real-time quantitative PCR experiments^42^. Regarding CCNB2 and CDK1, a recent study with primary HCC tissue samples showed that the downregulation of CCNB2 and CDK1 led to the inhibition of cell proliferation and cell cycle shutdown in the G2/M phase, indicating that the overexpression of CCNB2/CDK1 may promote tumour cell proliferation^14^. The experimental results of western blotting and real-time PCR showed that NDC80 contributed to HCC progression by reducing apoptosis and overcoming cell cycle termination^43^. Both BUB1B and TPX2 are associated with the separation of sister chromatids, which is the most abnormal phase in the progression of HCC^44^. In support of the accuracy of the Fisher score algorithm in predicting prognosis, Kaplan–Meier survival analysis revealed that the overexpression of the selected hub genes was correlated with reduced survival time.

WGCNA, a systems biology method for the analysis of correlation patterns among genes, has been heavily used in the field for hub gene identification. Based on a PubMed literature search, we found that more than 6000 WGCNA-related studies have been published so far. This finding demonstrated that WGCNA is a dominant method and is popular among researchers. In the current study, the Fisher score was demonstrated to be a method with similar performance to that of WGCNA, providing another viable methodological option in the field of hub gene identification. Random forest, with similar performance to that of WGCNA and the Fisher score, may also serve as a potential method for hub gene identification. In contrast, the Lasso and ReliefF algorithms do not seem to be good methods for hub gene identification, since the survival analysis showed that most of the hub genes identified by these methods were not relevant to the poor prognosis of HCC. The reason for the poor performance of Lasso and ReliefF may be that they randomly select one gene from correlated genes, which results in unstable performance in feature selection^24^.

In summary, we established an HCC dataset of a relatively large sample size by integrating five independent HCC datasets and demonstrated that the Fisher score algorithm is a suitable and accurate method for feature selection, thus providing an excellent option for hub gene identification in HCC patients.

## Methods

### Selection of datasets

A total of 31468 HCC expression arrays were available in GEO^45^. Only 5 (0.0159%) of the datasets were selected. The number of datasets was determined based on the following considerations. On the one hand, the integration of multiple datasets is helpful in fighting against the curse of dimensionality for feature selection in gene expression data. On the other hand, our study indicated that 5 datasets were sufficient for the identification of valid hub genes, and any further increase in datasets did not seem to be necessary.

Although the selected datasets were obtained from the same microarray platform, the microarray platform was only one of the criteria for data selection. Since this study mainly aimed to develop an effective method for the identification of HCC hub genes, only liver tissue datasets of Homo sapiens were considered. In addition, the sample size and data acquisition time were also important criteria for data selection. The datasets utilized in this study were obtained within the last 6 years with sample sizes greater than 40. Based on the above criteria, five datasets were selected in the current study. Due to a lack of techniques for RNA-Seq data analysis in our lab, RNA-Seq data were not included in this study.

The microarray gene expression profiles (GSE41804, GSE69715, GSE90653, GSE98383, and GSE107170) were downloaded from the GEO repository (http://www.ncbi.nlm.nih.gov/geo/). The platform information of these microarray data is as follows: GPL570, Affymetrix Human Genome U133 Plus 2.0 Array (Affymetrix Inc., Santa Clara, CA, USA). Since these observations were obtained on the same platform, these series of gene expression data share the same probe ID. All the files were integrated based on their probe ID.

### Batch effect identification and correction

The integrated microarray gene expression data originated from researchers of independent institutes. Therefore, there may be a batch effect that can cause a decrease in the repeatability and reproducibility of the experimental results. To detect the possible batch effects, PCA was performed to identify the batch effect. The batch effect was eliminated with the removeBatchEffect function of the limma package^46^. The visualization of the top two PCA components was assessed before and after the batch effect correction.

### Feature selection using the Fisher score algorithm

Before the application of the Fisher score algorithm^21^, the Affymetrix probe set IDs were converted to official gene symbols. Affymetrix probe set IDs without official gene names or corresponding to multiple official gene names were omitted. If multiple gene IDs corresponded to one official gene name, the expression value of the official gene was taken from the mean expression value of multiple gene IDs.

The Fisher score algorithm is a feature ranking algorithm applied to eliminate the irrelevant and redundant features from the gene expression profiles. The process of feature selection can be briefly described as follows. Assuming NG = (*U, C, D, δ*) is a neighbourhood decision system for gene expression data, the corresponding matrix is X ∈ R^*m×n*^, where m represents the number of genes, and n represents the number of samples. Then, the Fisher score is computed by

$$f(Z)=\frac{{\rm{tr}}({{\rm{A}}}_{b})}{{\rm{tr}}({{\rm{A}}}_{w})}$$

where tr () represents the trace of a matrix, A_w_ is the scatter matrix within the same category, and A_b_ is the scatter matrix between the HCC samples and their paired normal controls. To address the prolonged issue of traditional combination optimization methods, a heuristic strategy is normally utilized to calculate a score for each gene separately by using some criteria. Then, the Fisher score of the *l*-th gene is calculated by

$$f(k)=\frac{{\sum }_{k=1}^{C}\,{n}_{k}{({\mu }_{k}^{l}-{\mu }^{l})}^{2}}{{\sum }_{k=1}^{C}\,{n}_{k}{({\sigma }_{k}^{l})}^{2}}$$

where n_*k*_ represents the sample number of the *k*-th category, ${\mu }_{k}^{l}$ and ${\sigma }_{k}^{l}\,$ are the mean and standard deviation of the samples from the *k*-th category corresponding to the *l*-th gene, respectively, and *μ*^*l*^ represents the mean of the samples of the *l*-th gene.

### GO and KEGG analysis

GO analysis, a regular method in the annotation of large-scale functional enrichment studies, is normally classified into MF, BP, and CC categories^27^. KEGG^27^ is a widely utilized database for diseases, drugs, genomes, chemical substances and biological pathways. The GO and KEGG enrichment analysis of the selected feature genes in this study was performed using the Database for Annotation, Visualization and Integrated Discovery (DAVID) (https://david.ncifcrf.gov/) online tools^47,48^. P values less than 0.05 and gene counts more than 10 were considered statistically significant.

### GSEA

GSEA is a computational method that functions to identify classes of genes that are overrepresented in a large set of genes that may have a connection with disease phenotypes^28^. In this study, to further evaluate the performance of the Fisher score algorithm in selecting feature genes, the raw data of the integrated HCC gene expression data were applied in GSEA based on two major collections of MSigDB gene sets: hallmark gene sets and GO gene sets^49^. Hallmark genes were used since the hallmark gene sets can reduce redundancy and generate more robust enrichment analysis results.

### Establishment of a PPI network and identification of hub genes

The significantly enriched genes in the GO BPs were employed to establish the PPI network using the online PPI establishment tool STRING (http://string-db.org). The PPI data were then exported to Cytoscape version 3.4.0 (http://cytoscape.org)^50^. CytoHubba, a Java plugin for Cytoscape, provides a user-friendly interface that enables the topology analysis of complex networks^29^. CytoHubba provides 11 topological algorithms for identifying network hub genes. Among all the algorithms, MCC has a better performance in predicting PPI network hub genes^29^. Therefore, the MCC algorithm was employed to identify the HCC hub genes in this study.

### Survival analysis

The Kaplan–Meier plotter (KMplot, http://www.kmplot.com/analysis) can be employed to assess the effect of 54675 genes on survival with 10293 cancer samples^51^. The samples included in this database were obtained from 1648 ovarian, 5143 breast, 1065 gastric and 2437 lung cancer patients, with an average follow-up of 40, 69, 33, and 49 months, respectively^52,53^. The primary goal of this tool is to perform meta-analysis-based biomarker assessments. The HCC hub genes in this study were imported into the KMplot database to explore their relationship with the 5-year survival rates of patients.

### Identification of the hub genes with control algorithms

To further evaluate the performance of the Fisher score algorithm, a series of control feature selection algorithms were utilized to select feature genes from the current integrated HCC dataset. The algorithms for comparison included WGCNA^54^, Lasso^55^, ReliefF^56^ and random forest^57^.

To select the feature genes of HCC with the WGCNA algorithm, a series of procedures were carried out as follows. After loading the gene expression dataset, missing values and outlier microarray samples were checked to ensure that the data were appropriate for further analysis. A total of 3600 genes with the highest expression were then screened out based on the average gene expression value. We then selected the soft threshold using the network topology analysis function pickSoftThreshold. The gene expression matrix was then converted to an adjacency matrix and a topological overlap matrix (TOM). Hierarchical clustering of gene expression data was then performed based on the TOM-based dissimilarity distance. A dynamic tree cut function was employed to identify the modules (minimum module size = 30). GO and KEGG enrichment analysis was applied to select the HCC-related modules. Finally, the genes from the selected module were then used to construct a PPI network with the STRING database. The PPI network was visualized with Cytoscape followed by the identification of hub genes with the MCC algorithm.

For the identification of hub genes with Lasso, Relief and random forest, the procedure was identical to that of the Fisher score algorithm except that the feature selection algorithm was replaced with Lasso, Relief or random forest. We therefore only offered information related to feature gene selection according to these methods.

Regularization helps to address bias and variance as well as stabilize the estimates in a model. Lasso regression is one form of regularized regression. With the use of l1 regularizations, the coefficient of a variable can be reduced to zero. In the current Lasso approach for comparison, we used previously published methods by Regina *et al*.^55^ to select HCC feature genes. The feature gene selection procedures we followed are listed as follows. First, by tuning the parameter α, a list of genes with nonzero coefficients were selected with the Lasso. Second, the genes were sorted according to the absolute value of the coefficients in descending order. Third, the top 1000 genes of the sorted genes were selected for further analysis. The built-in Lasso algorithm in Scikit-learn (one of the machine learning libraries for the Python programming language) was utilized to select the feature genes from the current HCC dataset.

The ReliefF algorithm is a feature selection method proposed to handle multi-class classification problems that evaluates the importance of each feature by assessing the role of the features for classification between sample classes. By default, ReliefF assigns the same weight to each feature at the beginning. To score the weight for each feature, it randomly selects a sample T from a training set E and then finds the nearest neighbour sample B from the same class of sample T, called Near Hit; it then searches the nearest neighbour sample R from a class different from that of sample T, called Near Miss. Afterwards, it updates the weight of each feature according to the following rules. If the distance between T and Near Hit of a feature is less than the distance between T and Near Miss, this indicates that this feature is beneficial for distinguishing the nearest neighbours of the same class and different classes, so the weight of this feature will be increased. Conversely, if the distance between T and Near Hit is greater than the distance between T and Near Miss, this feature plays a negative role in distinguishing the nearest neighbours of the same class and different classes, and the weight of this feature will be reduced. The above process can be repeated m times, and finally, the weight of each feature is obtained. Here, the ReliefFAttributeEval function of Weka (version 3.83) was used to obtain the weight of all HCC genes in the current dataset, and the top 1000 weighted genes were screened out for further analysis.

For feature gene selection with random forest, the feature importance of feature X in the random forest was calculated as follows. First, for each decision tree in the random forest, the corresponding out-of-bag (OOB) data were used to calculate the OOB error, which is denoted as errOOB1. Second, random noise interference was added to the OOB data of feature X and the OOB data error was calculated once more, which is denoted as errOOB2. Third, assuming that there were N trees in the random forest, then the importance of the feature X = Σ (errOOB2-errOOB1)/N. In this way, random forests were performed that generate a list of all the variables based on their feature importance. Finally, the unimportant variables in the ranking list were deleted, leaving only the top 1000 important features. The RandomForestClassifier built-in in scikit-learn was applied for the feature selection of the current supervised classification HCC dataset.

The comparison among these methods is based on the prognostic value of the hub genes. A bioinformatics online tool (http://bioinformatics.psb.ugent.be/webtools/Venn/) was employed to obtain the intersections of the hub genes produced with the various approaches and to draw a Venn diagram. The hub genes were then also subjected to survival analysis with Kaplan–Meier plotter.

## Supplementary information

Supplementary figures

Dataset 1

Dataset 2
