# Supplementary material for: Feature selection with the Fisher score followed by the Maximal Clique Centrality algorithm can accurately identify the hub genes of hepatocellular carcinoma
Source: Sci Rep. 2019 Nov 21;9:17283. doi: 10.1038/s41598-019-53471-0 (PMC6872594; doi:10.1038/s41598-019-53471-0)
Supplement: Supplementary file 1 — Supplementary figures [file 41598_2019_53471_MOESM1_ESM.pdf]

Title: Feature selection with the Fisher score followed by the Maximal Clique Centrality algorithm can accurately identify the hub genes of hepatocellular carcinoma

Authors : Li Chengzhang, Xu Jiucheng

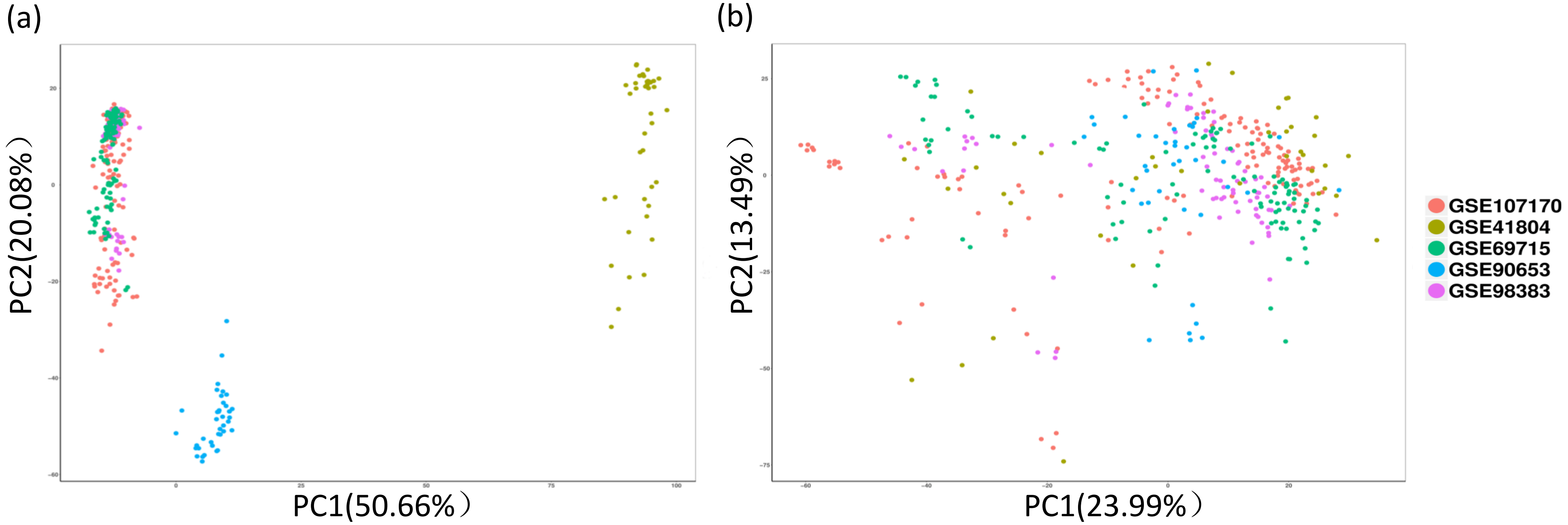
(a)
PC2(20.08%）
PC1(50.66%）
(b)
PC2(13.49%）
PC1(23.99%）
GSE107170
GSE41804
GSE69715
GSE90653
GSE98383

Supplementary Figure 1. Principal component analysis (PCA) of the integrated gene expression profiles. （a）PC1 and PC2 were the top 1 and 2 components, respectively, before the removal of the batch effect. A clear batch effect was identified prior to the batch effect correction. （b）PC1 and PC2 were the top 1 and 2 components, respectively, after the removal of the batch effect. The batch effect was eliminated after the batch effect correction.

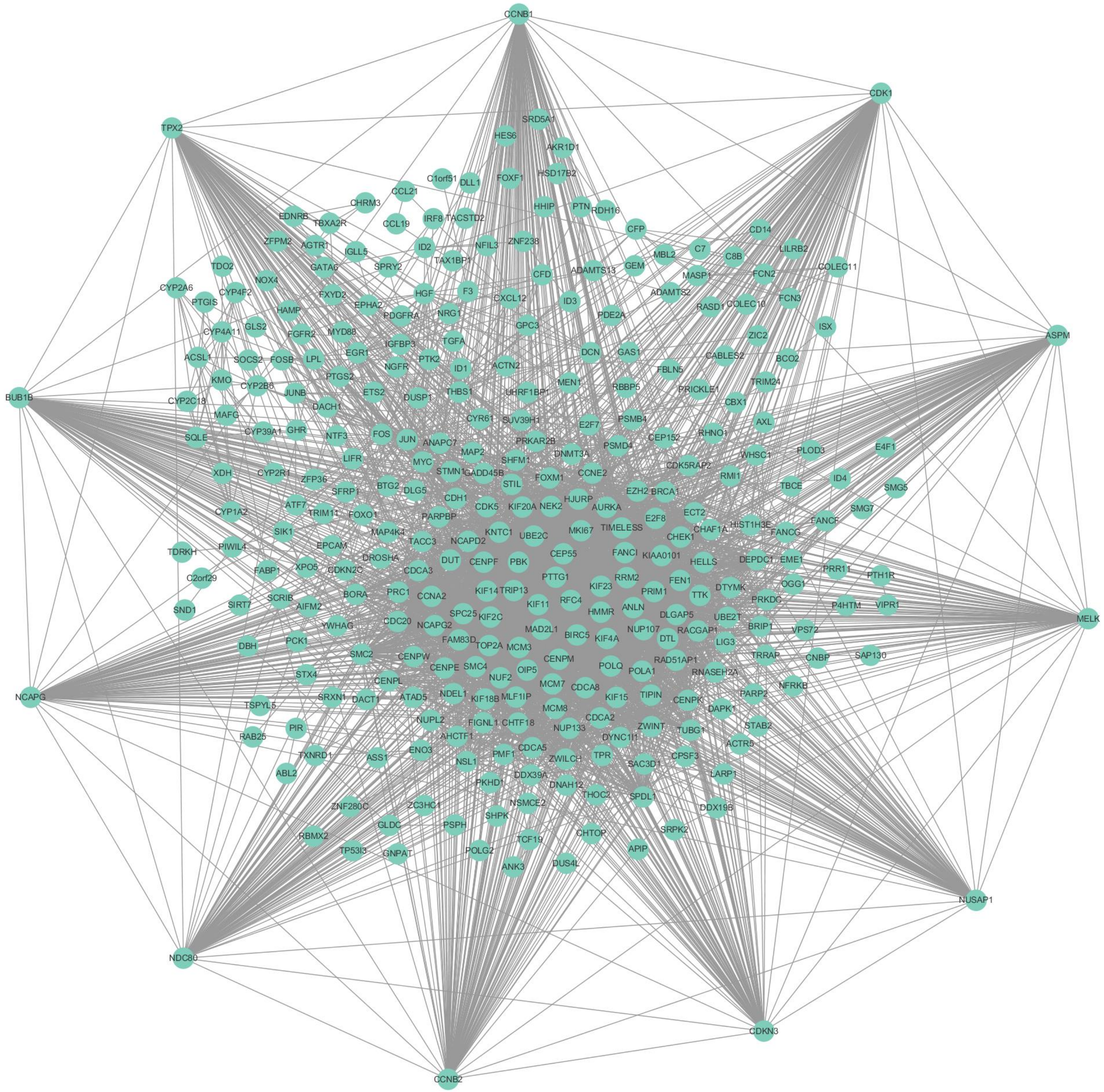
CCNB1
CDK1
TPX2
SRD5A1
HES6
AKR1D1
C1orf51
DLL1
FOXF1
HSD17B2
CCL21
CHRM3
HHIP
PTN
RDH16
EDNRB
TBXA2R
IRF8
TACSTD2
CCL19
CFP
CD14
ZFPM2
AGTR1
IGLL5
ID2
NFIL3
ZNF238
MBL2
C7
C8B
LILRB2
TDO2
GATA6
SPRY2
TAX1BP1
ADAMTS13
GEM
MASP1
FCN2
COLEC11
CYP2A6
NOX4
CYP4F2
CFD
ADAMTS2
PTGIS
HAMP
FXYD2
EPHA2
HGF
F3
CXCL12
ID3
RASD1
COLEC10
FCN3
PDGFRA
NRG1
PDE2A
ISX
CYP4A11
GLS2
FGFR2
MYD88
GPC3
ASPM
ACSL1
SOCS2
FOSB
LPL
EGR1
IGFBP3
TGFA
DCN
GAS1
CABLES2
ZIC2
BCO2
KMO
CYP2B6
PTGS2
NGFR
PTK2
ID1
ACTN2
FBLN5
BUB1B
JUNB
ETS2
DUSP1
THBS1
UHRF1BP1
MEN1
RBBP5
PRICKLE1
TRIM24
CYP2C18
DACH1
CBX1
MAFG
CYP39A1
GHR
NTF3
CYR61
SUV39H1
E2F7
PSMB4
AXL
SQLE
FOS
JUN
ANAPC7
PRKAR2B
CEP152
RHNO1
E4F1
MAP2
DNMT3A
PSMD4
PLOD3
LIFR
MYC
SHFM1
WHSC1
XDH
CYP2R1
STMN1
GADD45B
CCNE2
CDK5RAP2
RMI1
ID4
SMG5
ZFP36
STIL
FOXM1
TBCE
BTG2
DLG5
EZH2
BRCA1
SFRP1
CDH1
HJURP
ATF7
CDK5
KIF20A
NEK2
AURKA
SMG7
CYP1A2
TRIM11
FOXO1
PARPBP
E2F8
ECT2
FANCF
KNTC1
MKI67
TIMELESS
CHAF1A
HIST1H3E
FANCG
SIK1
MAP4K4
UBE2C
TACC3
NCAPD2
CHEK1
PIWIL4
EPCAM
CEP55
FANCI
KIAA0101
TDRKH
DROSHA
HELLS
DEPDC1
EME1
DUT
CENPF
PBK
FABP1
XPO5
CDKN2C
CDCA3
PRR11
PTH1R
C2orf29
PTTG1
RRM2
FEN1
DTYMK
OGG1
KIF14
TRIP13
KIF23
PRC1
PRIM1
BORA
CCNA2
RFC4
TTK
PRKDC
SIRT7
SCRIB
KIF11
P4HTM
VIPR1
AIFM2
SPC25
KIF2C
HMMR
ANLN
DLGAP5
UBE2T
SND1
CDC20
MAD2L1
NUP107
RACGAP1
MELK
YWHAG
NCAPG2
BIRC5
KIF4A
BRIP1
VPS72
DBH
PCK1
FAM83D
DTL
LIG3
TOP2A
MCM3
SMC2
CENPW
CENPM
RAD51AP1
TRRAP
CNBP
SAP130
CENPE
SMC4
POLQ
OIP5
POLA1
RNASEH2A
STX4
NUF2
CENPL
MCM7
CDCA8
NCAPG
NFRKB
PARP2
SRXN1
DACT1
ATAD5
NDEL1
KIF18B
MLF1IP
KIF15
TIPIN
TSPYL5
CENPK
MCM8
DAPK1
NUPL2
FIGNL1
CHTF18
CDCA2
PIR
RAB25
ZWINT
STAB2
NUP133
TUBG1
AHCTF1
DYNC1I1
ENO3
ACTR5
CDCA5
TXNRD1
ASS1
PMF1
ZWILCH
TPR
CPSF3
NSL1
SAC3D1
ABL2
DDX39A
LARP1
PKHD1
DNAH12
ZNF280C
ZC3HC1
THOC2
SPDL1
NSMCE2
DDX19B
SHPK
GLDC
PSPH
RBMX2
CHTOP
SRPK2
TCF19
POLG2
TP53I3
GNPAT
APIP
ANK3
DUS4L
NUSAP1
NDC80
CDKN3
CCNB2

Supplementary Figure 2. Protein-protein interaction network of the feature genes. The network has 374 nodes and 4478 edges. The nodes represent the selected feature genes, and the edges represent the interactions between two genes.
